# Case Report: Recurrent Kounis syndrome complicated with acute coronary stent thrombosis

**DOI:** 10.3389/fcvm.2026.1948247

**Published:** 2026-10-09

**Authors:** Yong Li, Chuanwen Jiang, Xiaojun Zhou, Qidong Cao

**Affiliations:** Department of Cardiology, People’s Hospital of Fengjie, Chongqing, China

**Keywords:** angiography, case report, coronary spasm, Kounis syndrome, stent thrombosis

## Abstract

**Background:**

Kounis syndrome manifests as an acute coronary syndrome induced by an allergic or hypersensitivity reaction. Kounis syndrome consists of three types: Type I is characterized by coronary artery spasm in normal or near-normal arteries, Type II by the rupture of coronary artery plaques, and Type III by the formation of thrombus within the coronary artery stent. Recurrent episodes of Kounis syndrome are uncommon in clinical practice. We herein report a case of recurrent Kounis syndrome, in which the second episode presented as Type III Kounis syndrome.

**Case presentation:**

We report a patient who experienced two acute coronary events triggered by Kounis syndrome within approximately one month—an uncommon presentation. One episode manifested as chest pain accompanied by tongue swelling, and was diagnosed as Type II Kounis syndrome and treated with cetirizine for anti-allergic purposes. The second episode occurred about one month later with persistent chest pain and recurrent tongue swelling, and coronary angiography confirmed acute coronary stent thrombosis. Taken together with the allergic manifestation (tongue swelling) accompanying both events, the second episode fulfilled the criteria for Type III Kounis syndrome. Subsequently, we performed emergency procedures to open the blocked coronary artery and administered glucocorticoid therapy. The patient's symptoms improved.

**Conclusion:**

Despite antihistamine therapy with cetirizine in the first episode, this patient still developed subsequent stent-related thrombosis. This case highlights that combined anti-allergic therapy including glucocorticoids may be considered at an early stage for Kounis syndrome patients with unidentified allergens, to mitigate potential allergic-mediated cardiac risk. Nevertheless, we cannot conclude glucocorticoids definitely prevent stent thrombosis, as avoidance of unrecognized allergens may also contribute to clinical improvement.

## 1 Introduction

Kounis syndrome (KS) manifests as an acute coronary syndrome (ACS) induced by allergic reaction (1). Based on coronary angiography findings, KS can be classified into three types: Type I involves normal coronary arteries; Type II refers to patients with pre-existing atherosclerotic plaques in the coronary arteries; and Type III involves thrombi within coronary stents (2). During an allergic reaction, allergic mediators trigger coronary artery spasm, directly cause plaque rupture, or induce intrastent thrombosis, thereby precipitating ACS (3).

KS seems not to be a rare disease. In a large-scale survey of hospitalized patients with allergic, hypersensitivity, or anaphylactic reactions in United States, 1.1% developed KS (4). However, it is infrequently reported in the literature and diagnosed in clinical practice, due to missed, unrecognized or non-diagnosed cases. Here we present a rare case of recurrent KS complicated by acute coronary stent thrombosis. We emphasize that combined anti-allergic regimens including antihistamines and glucocorticoids merit early consideration for patients without identifiable allergens. Although newer classification systems for KS have been proposed, we retained this traditional three-type classification for consistency with most existing clinical case reports.

## 2 Case introduction

A 67-year-old male was admitted with a 1-hour history of sudden chest pain accompanied by tongue swelling. The clinical timeline for the two episodes is summarized in Table 1. The patient had a 10-year history of hypertension and an 8-year history of diabetes mellitus, with a peak blood pressure of 180/100 mmHg. He took “Nifedipine tablets 10 mg twice daily” to control his blood pressure. He denied any relevant family-medical history, psychosocial risk factors, or genetic-related medical conditions. On physical examination, his body temperature 36.6 °C, pulse 64 beats/min, blood pressure 143/78 mmHg((1 mmHg = 0.133kPa). It was particularly noticeable that, the tongue was swollen to the extent that speech was difficult, just as the previous hospitalization about a month earlier. No urticaria, skin rash, pruritus, wheezing, stridor, or conjunctival oedema was noted. No other hypersensitivity-related physical signs were identified. Echocardiography showed ejection fraction of 52% and abnormal ventricular wall movement in the anterior and lateral walls of the heart. Routine blood tests, liver, kidney, blood lipid profiles were within the normal range. Myocardial injury markers were elevated significantly. The current admission ECG indicated acute anterior wall myocardial infarction (MI) (Figure 1).

**Table 1:** Timeline of two episodes of Kounis syndrome.

Time point	Clinical manifestations	Key examinations	Intervention
2025.09.04 (Episode 1)	Chest pain + tongue swelling	Angiography: LAD stenosis 70-95% + coronary spasm	LAD stent implantation Aspirin, clopidogrel, atorvastatin, cetirizine
2025.10.23 (Episode 2)	Recurrent chest pain + tongue swelling	ECG: acute anterior-wall MI; angiography: LAD in-stent occlusion	PTCA + drug-coated balloon in LAD Aspirin, clopidogrel, atorvastatin, cetirizine, prednisone added
2025.11.03 (Discharge)	Asymptomatic	—	Aspirin 100 mg qd, atorvastatin 20 mg qd, clopidogrel 75 mg qd, cetirizine 10 mg qd, prednisone 10 mg bid
6-month follow-up	No chest pain or tongue swelling	No recurrent events reported	Continued prescribed oral therapy

**Figure 1:**
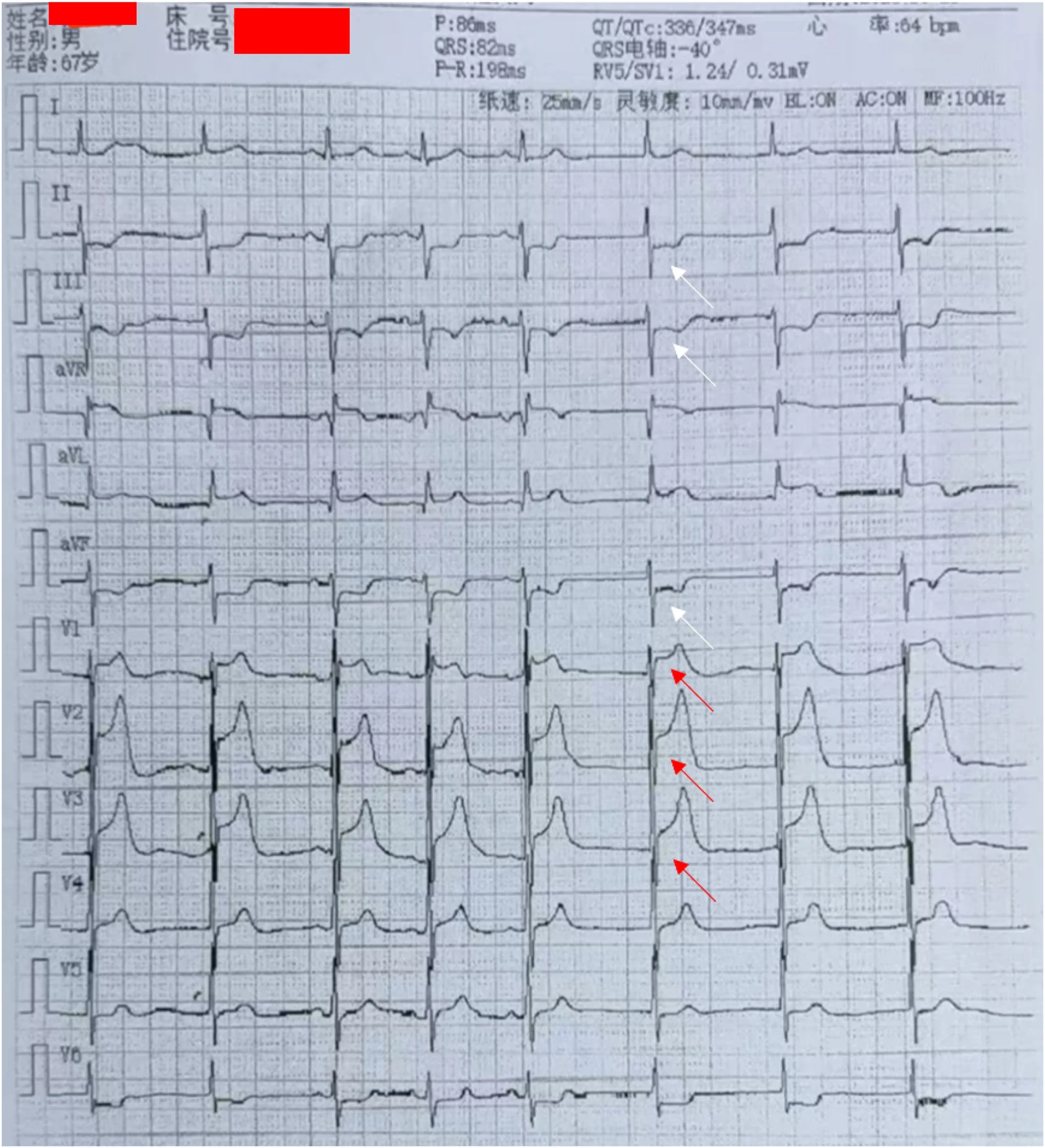
ECG showing ST segment elevation in leads V1-V3 (red arrow), with ST segment depression in the corresponding leads II, III, and avF (white arrow).

Immediate coronary angiography demonstrated an “intrastent occlusion in the left anterior descending artery (LAD)" (Figure 2A), establishing the diagnosis of acute ST-segment elevation myocardial infarction. After percutaneous transluminal coronary angioplasty, a drug-coated balloon was implanted (Figure 2B). Symptoms significantly improved, and the patient was admitted to the cardiac intensive care unit. He continued to receive antithrombotic and anti-allergic therapy including “aspirin, clopidogrel, atorvastatin and cetirizine” as last time. In addition, prednisone was added to suppress the underlying mast-cell-driven hypersensitivity cascade because recurrent Kounis-syndrome-related coronary events had occurred despite cetirizine monotherapy. The patient was instructed to be vigilant about possible allergenic substances.

**Figure 2:**
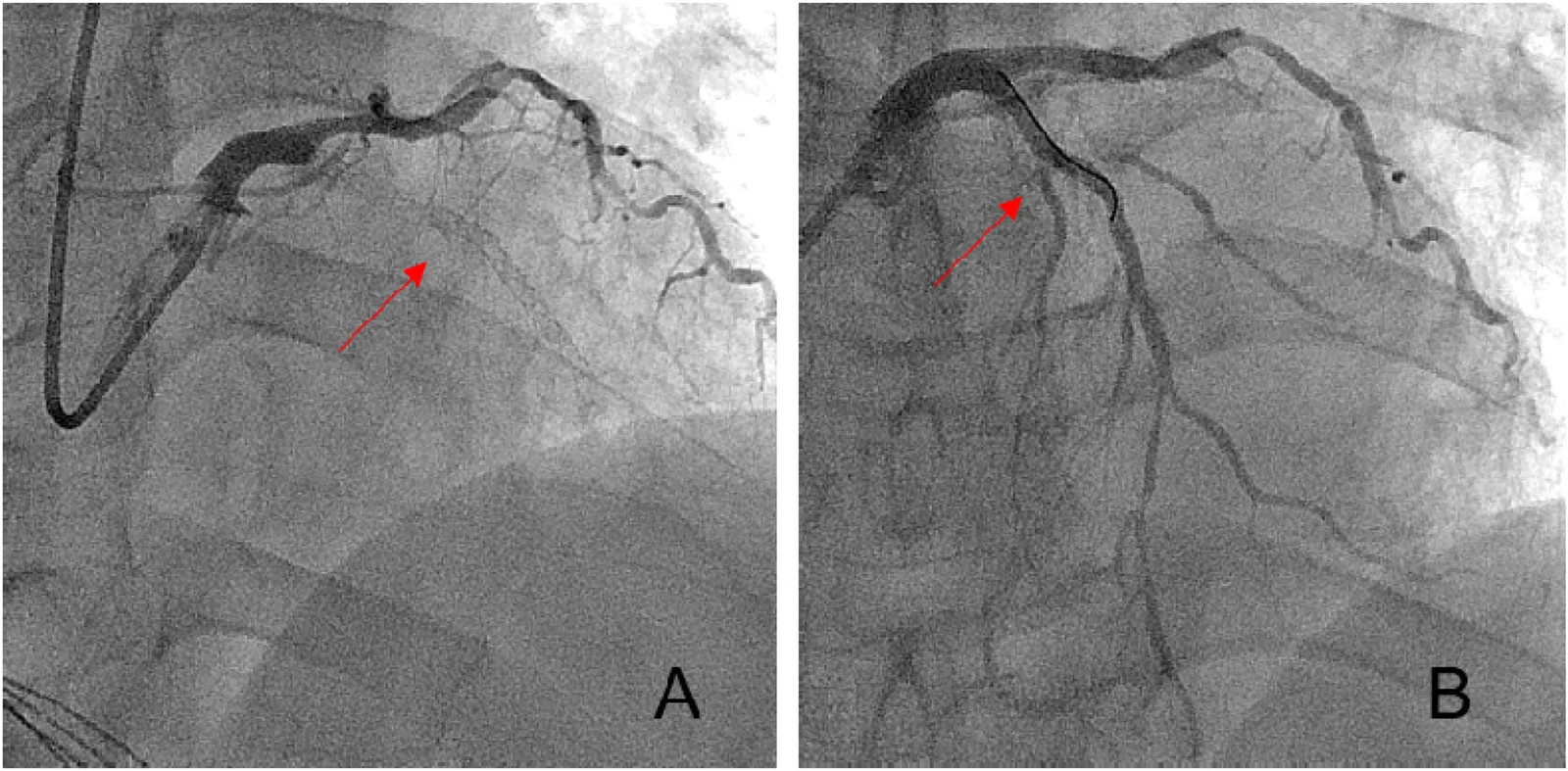
**(A)** Coronary angiography showing intrastent occlusion in the the proximal segments of the LAD (red arrow). **(B)** After percutaneous transluminal coronary angioplasty, anterograde TIMI grade 3 flow was restored and a drug-coated balloon was implanted (red arrow).

Interesting, about one month prior, the patient had presented to our hospital with similar symptoms. Coronary angiography revealed a “dominant right coronary artery pattern without significant abnormalities in the left main trunk, with diffuse long-segment lesions in the middle and distal segments of the LAD, showing stenosis of 70%–95% and spasm (Figures 3A,B).” Nitroglycerin 100 μg was given over and over again(the total amount is 200 μg) in the left coronary artery. Finally, the angiography showed relief of left coronary spasm with TIMI grade 3 (Figure 3C). A few days later, the patient underwent LAD stenting with two overlapping BioFreedom stents (2.25*24 mm and 2.75*24 mm) (Figure 3D). Standard balloon predilation and post-dilation were applied following routine interventional practice. The medications for treatment included “aspirin, clopidogrel, atorvastatin and cetirizine”.

**Figure 3:**
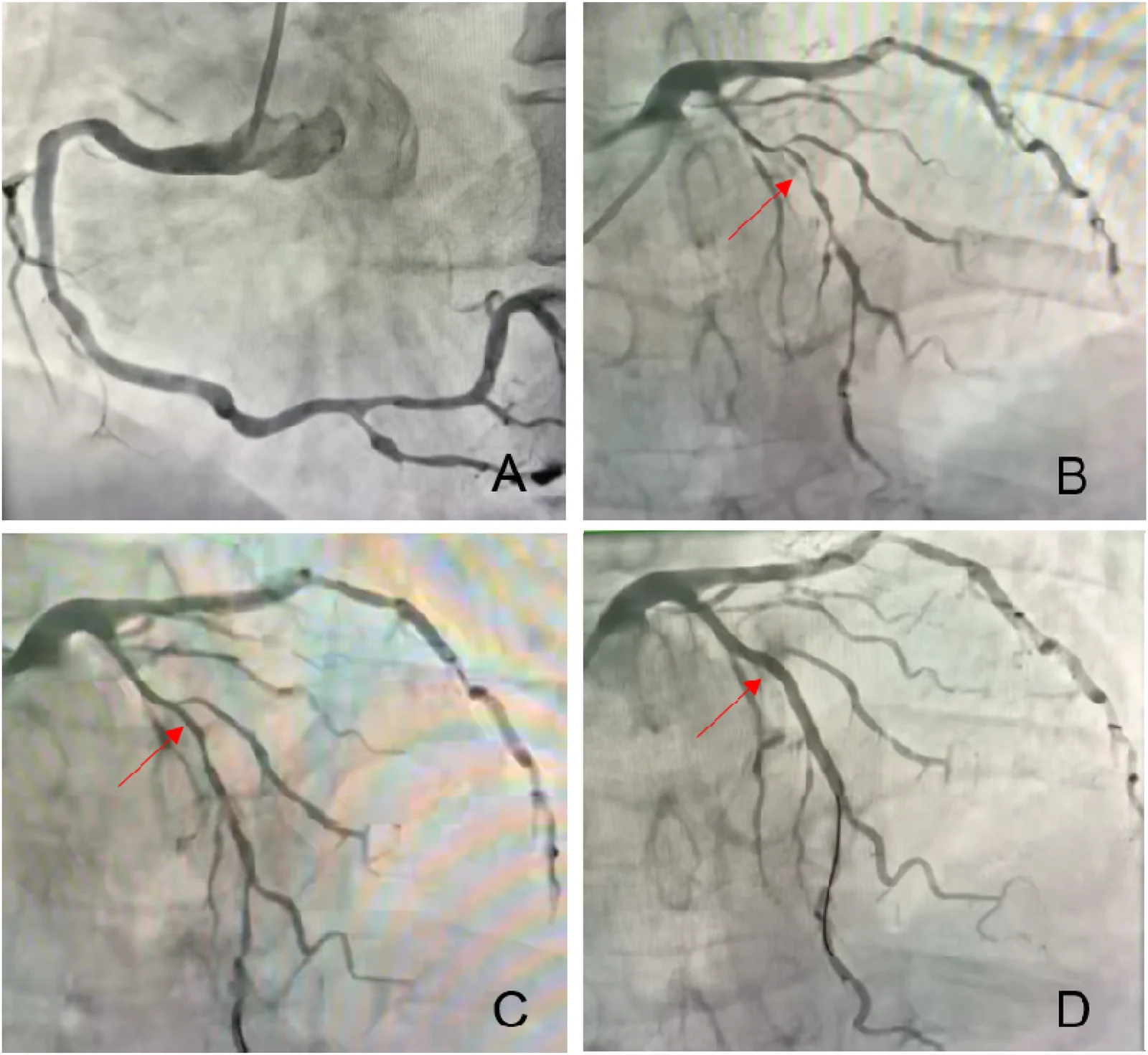
**(A)** The coronary angiography showing a tubular stenosis of approximately 30% in the middle segment of the right coronary artery. **(B)** Coronary angiography showing severe diffuse spasm of the middle and distal segments of the LAD with 70%–95% stenosis (red arrow). **(C)** The coronary angiography showing relief of spasm after intracoronary administration of nitroglycerin (red arrow). **(D)** The coronary angiography showing the significantly improved stenosis after the stent implantation (red arrow) in the LAD.

Given that the patient experienced two acute coronary events both accompanied by significant tongue swelling, recurrent KS was confirmed, and the second episode fulfilled diagnostic criteria for Type Ⅲ KS. The patient was discharged 10 days later. Medication after discharge: aspirin 100 mg once daily orally, atorvastatin 20 mg daily orally, clopidogrel 75 mg once daily orally, cetirizine 10 mg once daily orally, and prednisone 10 mg twice daily orally. During the subsequent 6-month follow-up period, patient was given education for long-term allergen avoidance and an emergency response plan for potential future hypersensitivity reactions. and he reported no discomfort or recurrence of symptoms. The patient was in stable condition without signs of cardiac or hypersensitivity-related complications on clinical evaluation. Therefore, repeated coronary angiography or additional laboratory testing was not performed during follow-up according to the patient's preference. The patient reported good adherence to all the prescribed oral regimen and adverse or unanticipated clinical events wasn^'^t occurred throughout the 6-month follow-up period.

## 3 Discussion

KS is an acute coronary syndrome caused by an allergic reaction, which can induce acute coronary artery events, first described by Kounis (5). when predisposed individuals are exposed to allergens, mast cells degranulate and release a large amount of inflammatory mediators, which can trigger coronary spasm, rupture of atherosclerotic plaques or formation of thrombus within the stent (6). Common allergens include various medications, foods, external environmental factors, etc (7). Unfortunately, the patient was unable to provide any specific food or drug exposure while repeated inquiries. Most studies suggest that KS involves mast cell activation via IgE-mediated and non-IgE-mediated pathways, releasing inflammatory mediators that trigger cardiovascular events (8).

Although there are no specific treatment standards for KS, glucocorticoids and H1 or H2 antihistamines are commonly used to alleviate allergic reactions (9). The patient initially developed unstable angina due to an allergic reaction and was treated with cetirizine. The patient underwent coronary stent implantation a few days later and was discharged from the hospital successfully. Unfortunately, the patient developed acute intrastent thrombosis about one month later, resulting in acute myocardial infarction (MI). Coronary angiography confirmed the diagnosis of types III KS, which was consistent with the majority of the reported cases of recurrent KS in the literature (10). Case reports by Ma et al. and Ferreira et al. demonstrated favourable event-free recovery under combined anti-allergic treatment in types III KS (11, 12). However, conventional causes of subacute stent thrombosis should be considered, such as stent underexpansion, stent malapposition, edge dissection, poor adherence to dual-antiplatelet therapy, and high on-treatment platelet reactivity. In the present case, intravascular imaging (IVUS/OCT) was not performed in either procedure, therefore mechanical-related stent complications cannot be fully ruled out. In addition, clopidogrel was selected as P2Y12 inhibitor mainly on the basis of the patient's comorbid profile and potential high bleeding risk. Mechanical thrombectomy was not performed during emergent revascularization for in-stent thrombosis, in accordance with guidelines suggesting that routine thrombus aspiration is not necessary when the thrombus burden is low. The patient experienced a recurrence of KS despite antihistamines treatment, culminating in a more severe MI, which suggested that the early combined use of antihistamines and glucocorticoids may be warranted in patients with KS without identifiable allergens. Furthermore, anti-IgE therapy with humanized monoclonal antibodies have been reported to be used for preventing the recurrence of KS (13, 14). However, such experience remains preliminary and derived mainly from individual case reports, and cannot be recommended as routine clinical therapy. For Type Ⅲ KS, potential underlying mechanisms include hypersensitivity to stent polymers, stent-coated drugs, or concomitant systemic medications. Crosstalk between mast cells, eosinophils and platelets jointly facilitates intrastent thrombus formation.

This case report still has certain limitations. First, critical allergy-related laboratory biomarkers were not collected during the two acute episodes, including serum tryptase (gold-standard biomarker for anaphylaxis), total/specific IgE, eosinophil counts and complement levels. Hence, allergic-mediated thrombosis remains a strong clinical hypothesis rather than laboratory-proven causality. Alternative contributors such as medication non-adherence, individual genetic susceptibility and procedural stent-related factors cannot be completely excluded. Second, we cannot fully attribute the six-month event-free follow-up exclusively to prednisone therapy, accidental avoidance of unknown causative allergen may also explain clinical remission. Third, intravascular imaging (IVUS/OCT) was absent, so mechanical stent-related causes for thrombosis could not be definitively excluded. Mechanical thrombectomy was not applied in the second-episode emergency procedure, which represents a procedural limitation. Last, formal patient-perspective interview was not documented, which is one shortcoming against CARE reporting guidelines. Anyhow, this rare real-world case presents sequential Type II and subsequent Type Ⅲ KS, both accompanied by objective systemic allergic manifestation (tongue swelling). It alerts clinicians that single H1-antihistamine monotherapy may be insufficient for preventing recurrent Kounis-related coronary events when causative allergens remain unidentified.

## 4 Conclusions

We report recurrent KS presenting first as Type II and, about one month later, as Type III (acute coronary stent thrombosis). Early combined anti-allergic therapy with antihistamines plus glucocorticoids may be considered when the allergen remains unidentified. However, evidence is limited to case reports. Prospective measurement of allergy biomarkers (serial tryptase, IgE, eosinophil count) and intracoronary imaging to exclude mechanical stent thrombosis are warranted in future cases. Nevertheless, we cannot conclude glucocorticoids definitely prevent stent thrombosis, as avoidance of unrecognized allergens may also contribute to clinical improvement.

## Data Availability

The original contributions presented in the study are included in the article/Supplementary Material, further inquiries can be directed to the corresponding author.
